# Draft Genome Sequence of Brucella ceti Isolated in the Western Pacific Ocean

**DOI:** 10.1128/MRA.00925-20

**Published:** 2020-09-24

**Authors:** Yuichi Ueno, Masahiko Kumagai, Hiroyuki Kanamori, Makio Yanagisawa, Sayuri Kino, Satoru Shigeno, Makoto Osaki, Daisuke Takamatsu, Ken Katsuda, Tadashi Maruyama, Kazue Ohishi

**Affiliations:** a National Institute of Animal Health, National Agriculture and Food Research Organization, Tsukuba, Ibaraki, Japan; b Advanced Analysis Center, National Agriculture and Food Research Organization, Tsukuba, Ibaraki, Japan; c Institute of Crop Science, National Agriculture and Food Research Organization, Tsukuba, Ibaraki, Japan; d Okinawa Churashima Foundation, Kunigami-gun, Okinawa, Japan; e Okinawa Prefectural Institute of Animal Health, Uruma, Okinawa, Japan; f The United Graduate School of Veterinary Sciences, Gifu University, Gifu, Gifu, Japan; g School of Marine Biosciences, Kitasato University, Sagamihara, Kanagawa, Japan; h School of Engineering, Tokyo Polytechnic University, Atsugi, Kanagawa, Japan; University of Maryland School of Medicine

## Abstract

In 2018, Brucella ceti was isolated from a bottlenose dolphin from the western Pacific Ocean. Here, we report a draft genome sequence of the isolate BD1442 of sequence type 27, which is the only sequence type known to have been isolated from human clinical cases.

## ANNOUNCEMENT

*Brucella* spp. are Gram-negative, facultative, intracellular bacteria that cause brucellosis in marine and terrestrial mammals, including humans (1). Among the *Brucella* spp., Brucella ceti and Brucella pinnipedialis have been isolated mainly from cetaceans and pinnipeds, respectively, and are known as marine *Brucella* spp. (2, 3). Most marine *Brucella* sp. isolates have been isolated from European and North American waters, and strains were not isolated from marine mammals in Asian or Oceanian waters until the late 2010s (2, 4–6).

In 2018, B. ceti was isolated from a bottlenose dolphin with osteomyelitis in an aquarium in Japan (6). The dolphin had been captured off the Pacific coast of Japan in 2009. Because anti*-Brucella* sp. antibodies were detected in the dolphin serum sample collected on the day of arrival at the aquarium, the dolphin was considered to have been infected while in the ocean. The bacterium was isolated from biopsy samples from connective tissue around the infected lesion after 4 days of incubation at 37°C on tryptic soy agar supplemented with 1% glucose and 5% horse serum under ambient and 10% CO_2_-containing air conditions (6). An isolate designated BD1442 was determined to be B. ceti and was classified into sequence type 27 (ST27) by multilocus sequence typing (MLST) (6). Although most B. ceti strains have been classified into two clades (i.e., dolphin and porpoise clades) by MLST, strains of ST27 remain distinct from those two clades and are considered to be located on the border between B. ceti and B. pinnipedialis (1). Although the isolation of marine *Brucella* spp. from humans is rare, ST27 is known to be the only ST to have been isolated from human patients (7).

To date, only a few genome sequences are available for B. ceti ST27 (8). Here, we report a draft genome sequence of B. ceti strain BD1442. For the genomic DNA extraction, BD1442 was cultured by the same method as that used for bacterial isolation under 10% CO_2_-containing air conditions. The genomic DNA was extracted using the Puregene Yeast/Bact. kit (Qiagen, Germany) and sheared into approximately 450-bp fragments using an LE220 ultrasonicator (Covaris, USA) with a microTUBE (Covaris). A DNA library was prepared using the TruSeq Nano DNA low-throughput library preparation kit (Illumina, USA) using the DNA fragments, which were tagged using TruSeq DNA single indexes (Illumina). The fragment size and concentration were measured using a 2100 Bioanalyzer (Agilent, USA). Sequencing was performed using the Illumina MiSeq platform (300-bp paired-end reads) with a MiSeq reagent kit v3 (Illumina) using an input concentration of 10 pM with 5% PhiX.

Paired-end reads were trimmed for adapter sequences and low-quality bases using fastp v0.20.0 with the default settings (9), and minimum Phred quality score thresholds of 15 for a single base and 20 for the mean of 4-bp sliding windows were established. A total of 7,548,692 preprocessed reads (2.26 Gbp) were assembled using SPAdes v3.13.2 with the options of careful and cov-cutoff auto (10). The assembly yielded 94 contigs using k-mers of 21, 33, 55, 77, 99, and 127. Following the removal of contigs shorter than 200 nucleotides (nt), the length of the remaining 83 contigs was 3,369,492 nt in total, the *N*_50_ value was 124,135 nt, and the GC content was 57.2%. The average coverage was 670.4×. DFAST v1.2.4 (11) was used for genome annotation; the genome consisted of 3,155 coding sequences, 1 rRNA, and 51 tRNAs.

### Data availability.

The draft genome sequence of B. ceti strain BD1442 has been deposited in the DNA Data Bank of Japan (DDBJ) under the accession numbers BLZI01000001 to BLZI01000083. The raw sequence data are available under SRA accession number DRX226376. The BioProject accession number is PRJDB10122, and the BioSample accession number is SAMD00233783.
